# Explaining Marital Patterns and Trends in Namibia: A Regression Analysis of 1992, 2000 and 2006 Demographic and Survey Data

**DOI:** 10.1371/journal.pone.0070394

**Published:** 2013-08-15

**Authors:** Lillian Pazvakawambwa, Nelago Indongo, Lawrence N. Kazembe

**Affiliations:** 1 Department of Statistics and Population Studies, University of Namibia, Windhoek, Namibia; 2 Multidisciplinary Research Centre, University of Namibia, Windhoek, Namibia; New York State Museum, United States of America

## Abstract

**Background:**

Marriage is a significant event in life-course of individuals, and creates a system that characterizes societal and economic structures. Marital patterns and dynamics over the years have changed a lot, with decreasing proportions of marriage, increased levels of divorce and co-habitation in developing countries. Although, such changes have been reported in African societies including Namibia, they have largely remained unexplained.

**Objectives and Methods:**

In this paper, we examined trends and patterns of marital status of women of marriageable age: 15 to 49 years, in Namibia using the 1992, 2000 and 2006 Demographic and Health Survey (DHS) data. Trends were established for selected demographic variables. Two binary logistic regression models for ever-married versus never married, and cohabitation versus married were fitted to establish factors associated with such nuptial systems. Further a multinomial logistic regression models, adjusted for bio-demographic and socio-economic variables, were fitted separately for each year, to establish determinants of type of union (never married, married and cohabitation).

**Results and Conclusions:**

Findings indicate a general change away from marriage, with a shift in singulate mean age at marriage. Cohabitation was prevalent among those less than 30 years of age, the odds were higher in urban areas and increased since 1992. Be as it may marriage remained a persistent nuptiality pattern, and common among the less educated and employed, but lower odds in urban areas. Results from multinomial model suggest that marital status was associated with age at marriage, total children born, region, place of residence, education level and religion. We conclude that marital patterns have undergone significant transformation over the past two decades in Namibia, with a coexistence of traditional marriage framework with co-habitation, and sizeable proportion remaining unmarried to the late 30s. A shift in the singulate mean age is becoming distinctive in the Namibian society.

## Introduction

Marriage and family formation are the cornerstone of every society, but are based on individuals choice and preferences which are significantly shaped by societal norms [1]. Marriage patterns vary considerably across countries and over time [1]–[10]. Such dynamics are also being experienced in African societies. Many studies, based on census and surveys, however, put emphasis on trends that have occurred. However, more important is to offer explanations to differences among individuals and trends over time. This paper aims to examine marital trends and patterns in Namibia and offer explanations to what has emerged since attaining independence in 1990. Particularly, we use national surveys based on the demographic and health surveys (DHS) data of 1992, 2000 and 2006 to establish factors that explain marital status patterns and trends. We hypothesize that there is considerable regional variation in marital status because of diverse cultural or ethnic differences in Namibia. We further postulate that education, religiosity, socio-economic status and place of residence are such factors that explain differences in marriage patterns among individuals and trends over time [4]–[10].

Research in understanding and modeling the formation of marriages and marital dissolutions has increased [4]. Nevertheless this area of research is old and global, and the processes that define nuptiality processes are multifaceted with social, behavioural and demographic factors which simultaneously influence marital status and trends [2]–[4]. Studies in the sub-Saharan Africa, Arab world, America, and Europe showed that women are staying single longer, or not marrying at all because of high costs associated with marriage, improved gender roles, educational expansion and secularization [5]–[9]. However, in many African societies, marriage is almost universal and early [5]. Within the same context, the percentages of marriages where both husband and wife are uneducated are decreasing [6]. An analysis of data on young American men and women from the National Longitudinal Survey of Youth from 1979 to 1992 showed that high earning capacity increased the probability of marriage and decreased the probability of divorce for young men [7]. In Europe, marriage patterns have mainly been explained by educational expansion, employment, secularization and changing gender roles [8]. An equivalent study in Norway reported that a large proportion of cohabiters indicated economic reasons for their hesitation to marry and in particular the costs of the wedding [9].

Recently, a considerable bulk of demographic research has reported increased cohabitation in Western and developed countries, with notable prevalence in developing countries. Ermisch and Francesconi [10] examined the dramatic increase in cohabiting unions in Great Britain. They analyzed entry into first partnership, the stability of cohabiting unions and re-partnering after dissolution of cohabitation. The shift to cohabitation as the dominant mode of first partnership played an important role in the delay of first marriage and motherhood. In a study based on the 2001 Nepalese Demographic and Health Surveys, Coltabiano and Castiglioni [11] described a significant downward shift in age at marriage, and delayed celebrated marriage in case of cohabitation. Cohabitation is also a common form of union in many southern African societies. For example in Botswana and South Africa, it is considered as a transitional stage before a bride price is paid, as such a consensual union may exist [5]. In Namibia, national surveys indicate that one in five adults are in cohabitation, and the prevalence is increasing [2], [3]. However factors affecting such marital processes have not been examined.

Various other factors affecting marriage, singlehood, cohabitation or divorce have been explored. Steel et al. [12], [13] have examined the effect of parenthood on whether non-marital unions led to marriage or parting. Their findings showed that the proportion of cohabiting couples who married before a birth decreased and the risk of dissolution declined during pregnancy. Lehrer [14] indicated that early age at first marriage is known to be associated with high risk of divorce. Another study in Britain established that education was a key factor influencing the age of entry into first partnership and whether or not the respondent would experience pregnancy before forming the partnership. Furthermore, religiosity, experience of parental separation, and the geographical region of residence were more important in affecting the decision to cohabit rather than to marry directly [15].

With regards to marriage dissolution, Bracher et al. [16] analyzed structural and temporal predictors of marriage dissolution. Their results indicated that the risk of marriage dissolution increased dramatically over the lives of the respondents. Year of birth, and age at marriage provided the most parsimonious characterization of the temporal correlates of marriage dissolution. Characteristics that were fixed by the time of marriage dissolution were related to characteristics of the unfolding marriage itself; namely patterns of employment, home-ownership, and region of residence. A review of research on the premarital factors associated with later marital quality and stability in first marriages was conducted by Larson and Holman [17]. Three major categories of factors were described including background and context, individual traits and behaviours, and couple interactional processes. They cited implications of their findings for family life education, premarital counseling and the need for further research.

The effect of such factors on the distribution and dynamics of intimate relationships in Namibia needs to be explored. The three survey data points spanning 1992 to 2006 present a unique opportunity to determine emerging trends and patterns in marital status. It is evident that not much research has been done to date, except in fertility studies [2]–[3], and in this study we focus exclusively on the association between different forms of unions and social-demographic variables. An attempt is also made to explore geographical variability in marital patterns in the country.

## Methods

### Data

This study is based on Namibian DHS of 1992, 2000 and 2006. DHS is a national survey drawn on using a multistage cluster sampling. At first stage, a random sample of enumeration areas (EA), which are primary sampling units, was chosen from the census sampling frame. From the selected EAs, households were systematically drawn. Only women of reproductive age (15–49 years), in the selected households, were interviewed using a face-to-face questionnaire. The questionnaire included variables on individual bio-demographic factors, household characteristics, history of marital unions and births. Final samples included in the analysis were respectively 5420 from the 1992 survey, 6755 from the 2000 survey and 9800 women, from the 2006/7 round of surveys.

### Outcome: Marital Status

All women involved in the survey were asked questions about their current marital status. The response was a multi-categorical variable of four categories: 1) never married, 2) married, 3) living together (co-habitation), and 4) others (widowed, separated and others). We also generated two binary outcomes: (i) coded 1 if ever married versus 0 if never married; and (ii) cohabiting versus married so that we assess the association between union formation, cohabitation and different socio-demographic covariates.

### Individual and household characteristics

Bio-demographic characteristics related to a woman included current age, age at first birth, age at first intercourse, total children born, employment status, religion and education level. Household characteristics consisted of region, place of residence (rural or urban) and wealth index. The region variable was recorded differently across the three surveys. The 1992 data recorded four broad geographic areas: northeast, northwest, central and south, while the 2000 and 2006 recorded 13 regions (Caprivi, Erongo, Hardap, Karas, Kavango, Khomas, Kunene, Ohangwena, Omaheke, Omusati, Oshana, Oshikoto, Otjozondjupa). To avoid errors introduced by aggregation/disaggregation we used the four regional groupings of 1992 in the subsequent analyses of all three waves of surveys. Again, 1992 data did not record a wealth index variable, but using the techniques as used for the 2000 and 2006 data we generated a wealth index for 1992. In brief, the wealth index was used as a proxy for the standard of living of the household. It is based on household ownership of consumer goods; dwelling characteristics; type of drinking water source; toilet facilities; and other characteristics related to household socio-economic status [20]. To construct the index, each of the assets was assigned a weight (factor score) generated through principal component analysis and the resulting asset scores were standardized in relation to the standard normal distribution. Each household was then assigned a score for each asset and the scores were summed for each household. Individuals were ranked according to the total score of the household in which they reside. The sample was then divided into quintiles from one (lowest) to five (highest) [18].

## Statistical Analysis

Firstly, we analyzed trends and computed percentage change in marital status between 1992 and 2006, particularly for the never married, married and living together categories by residence, age and education levels. We further investigated regional patterns in marital status by computing and mapping percentages and percentage changes for each category (never married, married and living together) using 2000 and 2006 data. This provides an exploratory analysis of the changes that have happened between 2000 and 2006, since similar geographical divisions were not available in the 1992 data.

Secondly, we examined the association between union formation (ever married) and socio-demographic variables using a binary logistic regression. We also explored if there was any shift towards cohabitation than marriage and investigated possible determinants by fitting a binary logistic regression. In a binary response, the variable 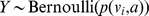 with
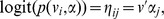
given some covariates 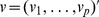 and corresponding parameter set 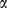. Third, we estimated factors that explained marital status by fitting a multinomial logistic regression model. A multinomial random variable applies where an event, 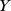, ends up with three or more outcomes 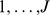 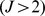. Specifically suppose 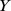 has unordered categories, we assume [19]

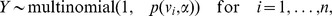
such that 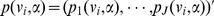, and 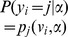, where 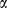 and 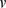 are as defined before. The most common approach to estimate multinomial probabilities is through the logistic model
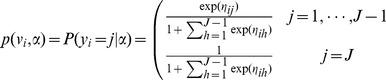
where 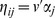 is the linear predictor. The last category 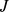 is considered as a reference classification outcome. In this classical multinomial logit model all covariates are assumed to be independent of the category while effects are category-specific.

The binary logistic regression models for ever married (vs single) and cohabitation (vs marriage) were fitted by combining all the data between 1992 and 2006. For the multinomial logistic, we fitted three models for the years 1992, 2000 and 2006 respectively. The last multinomial category (others) was assigned as a reference category. Put differently, we examined the likelihood of being: 1) never married versus others, 2) currently married versus others, and 3) currently living together (cohabitation) versus other forms of marital status. A p-value less than or equal to 0.05 was considered as significant. In all models, current age of the woman, age of the woman at first intercourse, age of the woman at first birth, and total number of children ever born to the woman were estimated as continuous variables, while region, education, religion, employment, wealth index and residence were estimated as categorical independent variables. Maximum likelihood estimation was applied. All models were fitted in SPSS version 19.

## Results

### Trends in marital status

Over half of the women interviewed in all the years were never married (50.0% in 1992, 50.3% in 2000 and 56.6% in 2006). This showed an increase of 6.6% for the period 1992–2006. The proportion married which consisted of 29.0% in 1992, 23.4% in 2000 and 20.4% in 2006, declined by 8.4% in the same period. Women who were cohabiting with their partners were 13.4% in 1992 and this increased to 18.4% in the 2000 survey, but dropped 16.0% in 2006. The other forms of marital status showed a slight downward change between 1992 and 2006. A chi-square test for trend showed a significant change in marital patterns in Namibia between 1992 and 2006 (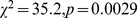).

Table 1 shows bivariate associations between never married, ever married, cohabitation and background characteristics. The percentage ever married shows a decline while cohabitation shows an increase in association with age (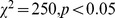) and between 1992 and 2006 (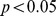). There was also evidence of differences in proportion ever married and cohabiting by region, residence and education level (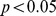).

**Table 1:** Bivariate associations of never married, ever married and cohabitation by background characteristics.

Variable	Category	Never married	Ever married	Cohabiting	Total (*n*)
Year	1992	50.0	50.0	31.6	5421
	2000	50.3	49.7	44.0	6755
	2006	56.6	43.4	44.0	9804
Education	None	25.2	74.8	48.3	2370
	Primary	47.5	52.5	44.9	7462
	Sec/higher	61.8	38.2	34.5	12148
Religion	Protestant	54.2	45.8	38.6	16762
	Catholic	49.2	50.8	47.2	5218
Employment	Unemployed	58.8	41.2	43.6	12486
	Employed	45.4	54.6	37.8	9399
Residence	Rural	53.4	46.6	39.1	12588
	Urban	52.5	47.5	42.9	9398
Wealth index	Poorest	55.0	45.0	41.7	4139
	Poor	50.8	49.2	49.8	4526
	Medium	54.9	45.1	50.9	5054
	Rich	53.6	46.4	45.8	3857
	Richest	50.3	49.7	20.0	4044
Region	Northwest	66.5	33.5	35.1	8021
	Northeast	39.2	60.8	33.1	3878
	Central	45.7	54.3	52.0	5087
	South	49.5	50.5	40.7	4994
Age	15–19	92.5	7.5	68.7	4926
	20–24	70.1	29.9	62.8	4325
	25–29	49.9	50.1	51.4	3548
	30–34	33.4	66.6	38.8	3149
	35–39	24.8	75.2	33.2	2412
	40–44	19.7	80.3	27.4	2111
	45–49	15.1	84.9	22.7	1509

Given are percentage unless stated.

**Figure 1:**
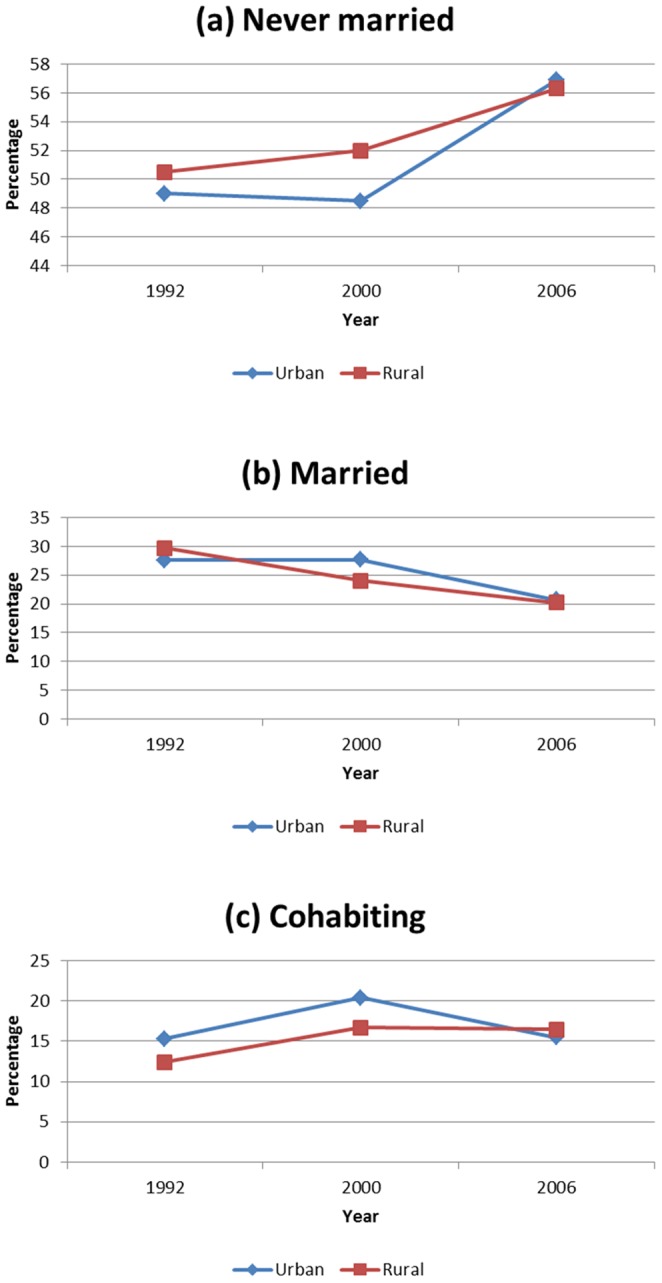
Trends in marital status. (a) never married; (b) married and (c) cohabiting between 1992 and 2006 by rural/urban place of residence.

**Figure 2:**
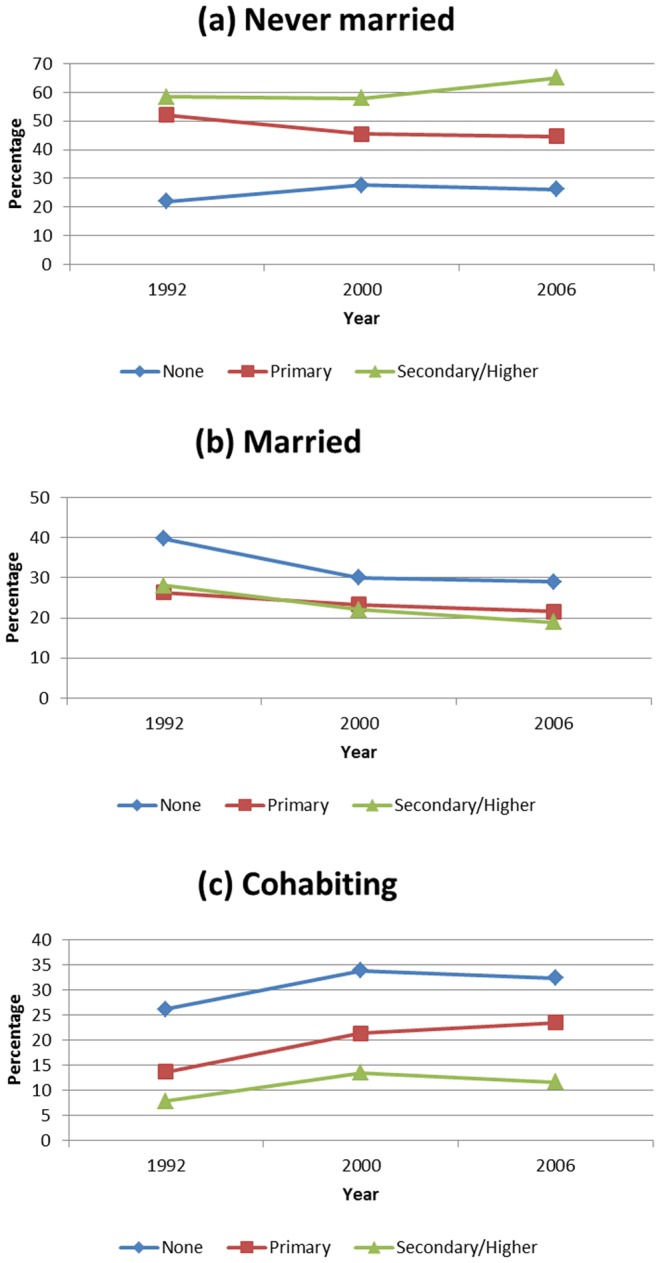
Trends in marital status. (a) never married; (b) married and (c) cohabiting between 1992 and 2006 by education level.

**Figure 3:**
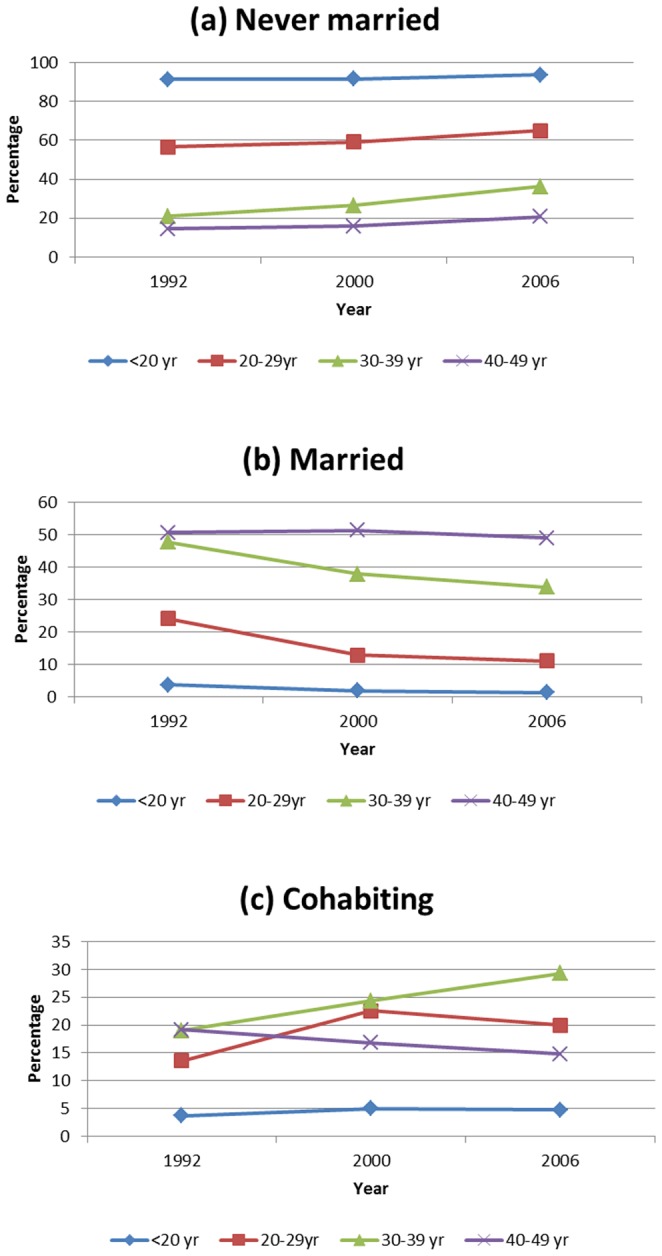
Trends in marital status. (a) never married; (b) married and (c) cohabiting between 1992 and 2006 by current age.

Next, we explored changes in trends by residence, age and education. Figures 1 to 3 give results. Among the never married, the proportion in the urban areas was relatively lower than in the rural areas between 1992 and 2000, but this changed in 2006 (Figure 1a). Similar trend was observed among the married (Figure 1b). For the living together, the proportion was higher in urban areas than rural areas, with increased trends for both areas between 1992 and 2000. This pattern changed in 2006, with relatively more partners living together in rural than urban areas (Figure 1c).

With regards to education level, the never married were relatively higher in proportion among those who attained secondary or higher education than among those with primary education or none. This higher trend persisted for all the years (Figure 2a). However, among the married women those with primary education were relatively more, followed by those without any formal education. Lower proportions of marriage were observed for those who achieved secondary or higher education levels and the trend was steady for the years (Figure 2b). The pattern observed among those living together was similar to those who were married (Figure 2c).

**Figure 4:**
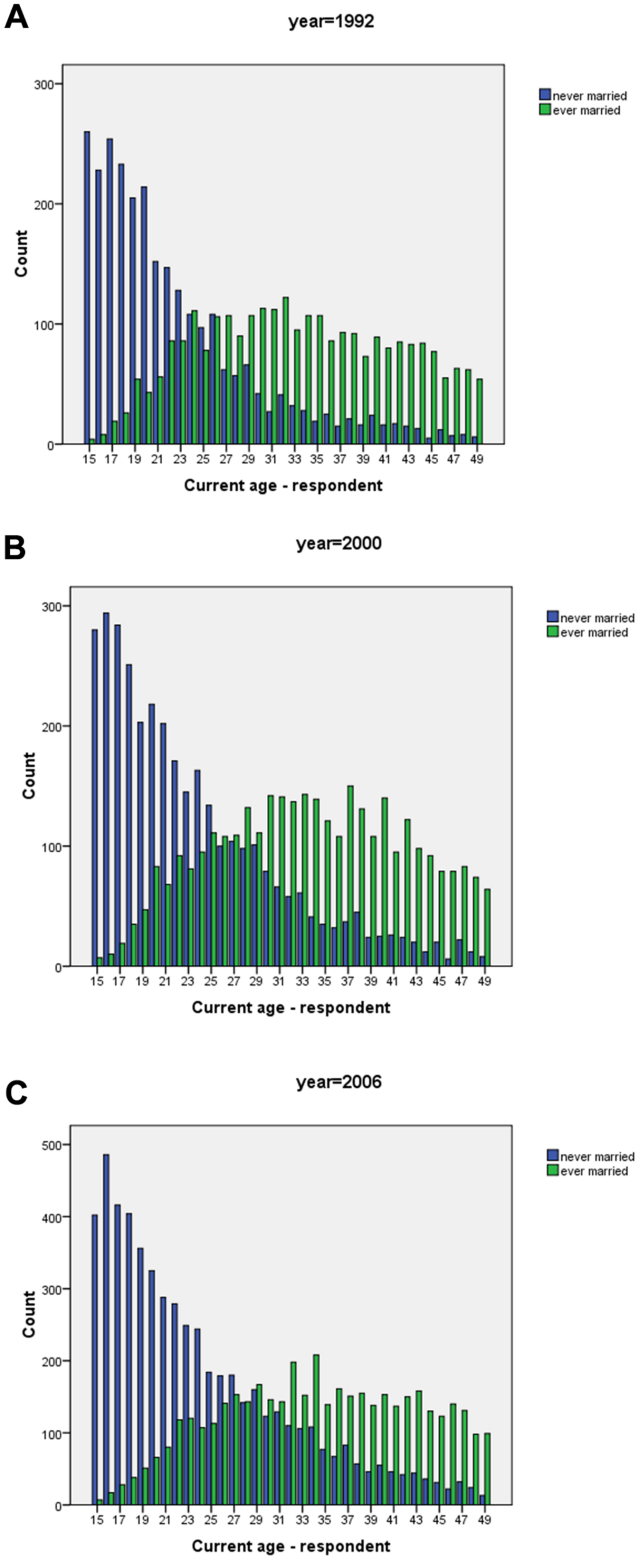
Cumulative number of women aged between 15–49 ever married or never married for: (a) year 1992; (b) year 2000 and (c) year 2006.

Turning to the effect of age on the trend of marriage patterns, we observed as expected that those below age 20 mostly remaining unmarried with decreasing percentages as age increased (Figure 3a). In Figure 3b, as expected, we had a reverse in trends among the married, with increased rates as age increased persistently from 1992. A mixed pattern was notable among those living together, however, the proportion cohabitating with age below 20 years remained lower than 5% from 1992 (Figure 3c). However overall, there is a shift in modal age at marriage between 1992 and 2006 (Figure 4). In 1992, this was at 23 years, while in 2000 this shifted to 27 and as of 2006 this was at 29 years of age. This is in agreement with what has been discussed before (Figures 1 to 3).

### Geographical patterns in marital status

**Figure 5:**
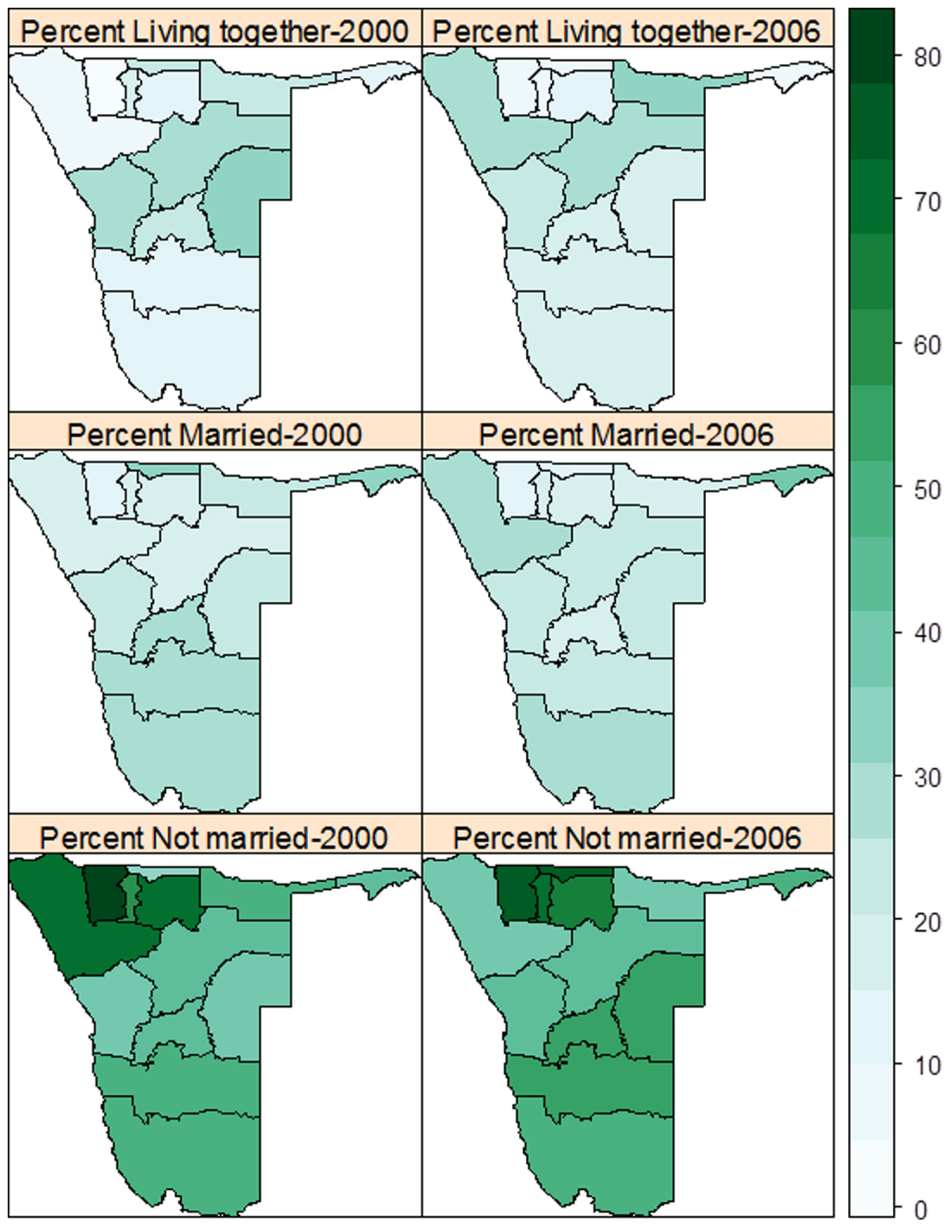
Regional proportions in marital status (never married, married and living together) in 2000 and 2006.

**Figure 6:**
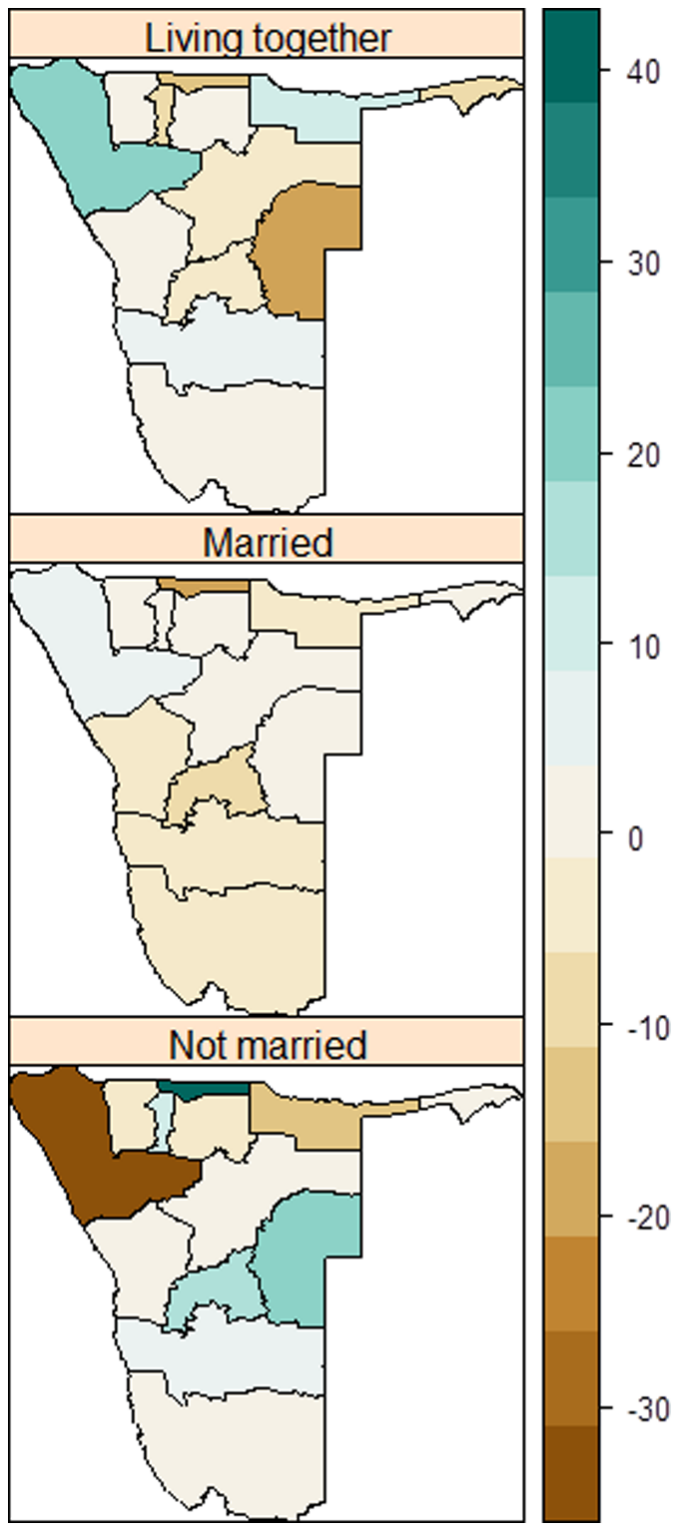
Percentage changes in marital status by region between 2000 and 2006 in Namibia.

Figure 5 shows regional variation in marriage patterns. In 2000, a higher percentage living together was observed in the northern provinces of Kunene, Kavango, and Otjozondjupa, while in 2006 the highest rates were observed in central regions, particularly in Erongo, Khomas and Omaheke. For the married group, there were no significant differences across the regions for both 2000 and 2006 surveys, although Caprivi registered a relatively higher percentage married than other regions at both times. Significant regional differences were observed among the never married, particularly in the north and south (Figure 5, bottom panel). An assessment of percentage change in marital patterns shows a decline among the never married in Kunene, while an increase among the living together in the same region (Figure 6). A relative decline in percent married was registered in many regions, especially in the central and southern regions. Among those living together, an increased change was notable in Kunene and Kavango whereas a corresponding decline was observed in Omaheke (Figure 6).

### Ever in union and its determinants

Table 2 gives results of the model that predicts the probability of being in the union as a function of year, current age, region, education, religion, employment and residence. The probability of ever married was 33% higher in 1992 compared to 2006 but decreased to 29% in 2000. The proportion of women ever having been in a union increased with age, while the odds of ever been in a union was much higher in the North-east (OR = 2.31, 95% CI: 2.06, 2.59) and central (OR = 1.17, 95% CI: 1.06, 1.29) regions of Namibia compared to the south, but much lower in Northwest (OR = 0.45, 95% CI: 0.41, 0.49). Results further show that the chance of married was much higher for those with no formal education or lower education level than those who attained secondary or higher (OR = 2.47 and 1.70 respectively). The effect of employment was marginal and that of wealth index was largely non-significant. Finally the effect of urban residence was lower, as expected, for the ever in union group compared to those in rural areas.

**Table 2:** Logistic regression results for ever in union (vs single) and cohabitation (vs marriage).

		Ever married		Cohabiting	
		vs Never married		vs Married	
Variable	Category	OR	95% CI	OR	95% CI
Year	1992	1.33	(1.22, 1.45)	0.54	(0.47, 0.61)
	2000	1.29	(1.19, 1.39)	0.81	(0.73, 0.91)
	2006	1.00		1.00	
Education	None	2.47	(2.18, 2.79)	2.24	(1.92, 2.61)
	Primary	1.70	(1.57, 1.84)	1.71	(1.52, 1.93)
	Sec/higher	1.00		1.00	
Religion	Protestant	1.19	(1.01, 1.29)	1.39	(1.25, 1.56)
	Catholic	1.00		1.00	
Employment	Unemployed	1.00		1.00	
	Employed	1.08	(1.00, 1.16)	0.95	(0.85, 1.05)
Residence	Rural	1.00		1.00	
	Urban	0.78	(0.72, 0.85)	1.93	(1.70, 2.18)
Wealth index	Poorest	1.00		1.00	
	Poor	1.01	(0.91, 1.13)	1.01	(0.86, 1.18)
	Medium	1.10	(0.98, 1.23)	1.16	(0.99, 1.35)
	Rich	0.94	(0.83, 1.07)	1.05	(0.88, 1.26)
	Richest	1.27	(1.10, 1.47)	0.27	(0.22, 0.34)
Region	Northwest	0.45	(0.41, 0.49)	0.71	(0.61, 0.82)
	Northeast	2.31	(2.06, 2.59)	0.38	(0.32, 0.44)
	Central	1.17	(1.06, 1.29)	1.21	(1.06, 1.39)
	South	1.00		1.00	
Age	15–19	0.013	(0.011, 0.016)	9.53	(7.06, 12.86)
	20–24	0.08	(0.06, 0.09)	8.01	(6.49, 9.87)
	25–29	0.19	(0.16, 0.23)	4.65	(3.83, 5.65)
	30–34	0.39	(0.33, 0.46)	2.55	(2.11, 3.07)
	35–39	0.58	(0.49, 0.69)	1.88	(1.55, 2.29)
	40–44	0.78	(0.65, 0.94)	1.44	(1.18, 1.76)
	45–49	1.00		1.00	

### Cohabitation and its determinants

Table 2 again shows factors associated with cohabitation union versus being in traditional marriage. As one can observe, the propensity of cohabitation increased by 50% between 1992 and 2000, and 85-fold between 1992 and 2006 and at same time by 23% between 2000 and 2006. However, cohabiting decreased with increasing age. In other words, most cohabitation unions ended up in formal marriage. We also noted that cohabitation was much higher in the central region compared to the south (OR = 1.21, 95% CI: 1.06, 1.39). This is the region where Windhoek, the capital city is located. What was surprising, though, from our findings is that being less educated was associated with increased probability of cohabitation compared to those who were of secondary or higher education levels (OR = 2.24 for no education and OR = 1.71 for primary education respectively). Furthermore, we observed that living in urban areas increased the chance of cohabitation compared to living in rural areas (OR = 1.93, 95%CI: 1.70, 2.18).

### Individual and household characteristics associated with never been married

Table 3 presents results from the multinomial regression model. Being single was significantly associated with current age, total children ever born, age at first birth and age at first sex. The odds of remaining single decreased with increasing age, number of children the woman borne, and with increased age at first sex, while being single increased with increasing age at first birth. The effect of region varied in between the years. Between 1992 and 2006, women were likely to remain single in the northeast and south compared to the northwest. For those from the central region, the likelihood of being single was lower in 1992, but higher in the year 2000 and 2006 compared to those in the northwest region.

**Table 3:** Multinomial regression results on marital status based on 1992, 2000 and 2006 DHS data.

		1992		2000		2006	
Variable	Category	RR	95% CI	RR	95% CI	RR	95% CI
***NOT MARRIED***							
Current Age		0.87	(0.85, 0.89)	0.91	(0.89, 0.93)	0.89	(0.88, 0.90)
Total children born		0.89	(0.82, 0.96)	0.85	(0.79, 0.92)	0.79	(0.74, 0.84)
Age at first birth		1.05	(1.01, 1.10)	1.03	(1.00, 1.06)	1.03	(1.00, 1.06)
Age at first intercourse		0.96	(0.95, 0.97)	0.98	(0.97, 0.99)	0.96	(0.95, 0.97)
Region	Northwest	1.00		1.00		1.00	
	Northeast	25.8	(17.33, 38.39)	1.72	(1.18, 2.51)	7.72	(5.84, 10.22)
	Central	0.74	(0.37, 1.48)	1.49	(1.11, 1.99)	2.27	(1.72, 3.01)
	South	3.05	(1.85, 5.02)	1.49	(1.08, 2.06)	1.71	(1.26, 2.32)
Education	None	1.00		1.00		1.00	
	Primary	0.82	(0.57, 1.20)	1.43	(1.00, 2.06)	0.89	(0.64, 1.23)
	Secondary or higher	0.77	(0.49, 1.20)	1.09	(0.74, 1.59)	0.71	(0.50, 1.01)
Religion	Catholic	1.00		1.00		1.00	
	Protestant	0.93	(0.69, 1.25)	0.83	(0.64, 1.06)	0.97	(0.78, 1.21)
Employment	Unemployed	1.00		1.00		1.00	
	Employed	1.38	(1.03, 1.85)	1.66	(1.31, 2.09)	1.25	(1.03, 1.53)
Wealth index	Least poor	1.00		1.00		1.00	
	Poor	0.99	(0.65, 1.50)	1.00	(0.72, 1.38)	0.95	(0.69, 1.29)
	Medium	1.18	(0.78, 1.78)	0.67	(0.47, 0.96)	0.86	(0.62, 1.20)
	Richer	1.37	(0.85, 2.22)	0.64	(0.44, 0.93)	0.79	(0.54, 1.16)
	Richest	1.19	(0.65, 2.17)	0.63	(0.39, 1.02)	0.81	(0.51, 1.30)
Residence	Rural	1.00		1.00		1.00	
	Urban	0.8	(0.54, 1.19)	1.29	(0.99, 1.70)	1.18	(0.92, 1.50)
***MARRIED***							
Current Age		0.94	(0.92, 0.96)	0.98	(0.97, 1.00)	0.95	(0.93, 0.96)
Total children born		1.23	(1.16, 1.32)	1.20	(1.12, 1.28)	1.25	(1.18, 1.32)
Age at first birth		1.09	(1.05, 1.13)	1.06	(1.03, 1.10)	1.10	(1.07, 1.12)
Age at first intercourse		1.00	(1.00, 1.00)	1.00	(1.00, 1.01)	1.00	(0.99, 1.00)
Region	Northwest	1.00		1.00		1.00	
	Northeast	1.39	(1.00, 1.94)	0.94	(0.65, 1.36)	1.01	(0.78, 1.31)
	Central	0.38	(0.19, 0.75)	1.27	(0.94, 1.70)	1.00	(0.75, 1.31)
	South	1.03	(0.64, 1.65)	1.01	(0.73, 1.39)	1.01	(0.75, 1.38)
Education	None	1.00		1.00		1.00	
	Primary	0.95	(0.70, 1.29)	1.54	(1.09, 2.17)	1.12	(0.83, 1.51)
	Secondary or higher	0.67	(0.45, 0.99)	1.17	(0.81, 1.69)	0.85	(0.61, 1.18)
Religion	Catholic	1.00		1.00		1.00	
	Protestant	1.08	(0.83, 1.41)	0.95	(0.74, 1.22)	0.62	(0.50, 0.77)
Employment	Unemployed	1.00		1.00		1.00	
	Employed	1.75	(1.35, 2.28)	1.55	(1.23, 1.95)	1.08	(0.89, 1.32)
Wealth index	Least poor	1.00		1.00		1.00	
	Poor	0.84	(0.58, 1.19)	1.00	(0.73, 1.37)	0.81	(0.60, 1.10)
	Medium	0.91	(0.64, 1.29)	0.78	(0.55, 1.11)	0.58	(0.42, 0.80)
	Richer	0.91	(0.59, 1.40)	0.78	(0.53, 1.14)	0.41	(0.28, 0.59)
	Richest	0.37	(0.21, 0.64)	0.15	(0.10, 0.24)	0.16	(0.10, 0.25)
Residence	Rural	1.00		1.00		1.00	
	Urban	1.59	(1.12, 2.26)	1.72	(1.31, 2.26)	2.01	(1.57, 2.56)
***LIVING TOGETHER***							
Current Age		0.91	(0.89, 0.93)	0.92	(0.90, 0.94)	0.89	(0.87, 0.90)
Total children born		1.08	(1.00, 1.16)	1.07	(1.00, 1.15)	1.12	(1.05, 1.19)
Age at first birth		1.05	(1.01, 1.09)	1.03	(1.00, 1.06)	1.04	(1.01, 1.07)
Age at first intercourse		0.99	(0.99, 1.00)	1.00	(1.00, 1.01)	0.99	(0.99, 1.00)
Region	Northwest	1.00		1.00		1.00	
	Northeast	8.79	(5.86, 13.20)	1.00	(0.67, 1.48)	1.18	(0.89, 1.57)
	Central	0.31	(0.16, 0.63)	0.79	(0.58, 1.07)	0.49	(0.37, 0.66)
	South	1.11	(0.67, 1.86)	1.19	(0.85, 1.68)	0.46	(0.33, 0.63)
Education	None	1.00		1.00		1.00	
	Primary	1.45	(1.03, 2.05)	2.19	(1.55, 3.09)	1.15	(0.85, 1.56)
	Secondary or higher	2.44	(1.55, 3.83)	2.68	(1.85, 3.90)	1.68	(1.20, 2.35)
Religion	Catholic	1.00		1.00		1.00	
	Protestant	1.12	(0.83, 1.53)	1.25	(0.97, 1.61)	1.25	(1.00, 1.55)
Employment	Unemployed	1.00		1.00		1.00	
	Employed	1.27	(0.95, 1.71)	1.71	(1.35, 2.17)	1.42	(1.15, 1.74)
Wealth index	Least poor	1.00		1.00		1.00	
	Poor	1.12	(0.73, 1.73)	0.71	(0.51, 0.99)	1.11	(0.80, 1.53)
	Medium	0.94	(0.62, 1.42)	0.43	(0.30, 0.62)	0.81	(0.58, 1.13)
	Richer	0.83	(0.51, 1.36)	0.51	(0.35, 0.76)	0.74	(0.50, 1.10)
	Richest	0.92	(0.49, 1.72)	0.77	(0.46, 1.30)	1.05	(0.64, 1.72)
Residence	Rural	1.00		1.00		1.00	
	Urban	1.13	(0.76, 1.67)	0.77	(0.58, 1.01)	1.26	(0.99, 1.62)

Education showed significant association with the never married in 2000, that is those with primary education were likely to remain single much longer than those with no education. No association with region was found, but being employed compared to being unemployed increased the likelihood of remaining single (RR = 1.38 in 1992, RR = 1.36 in 2000 and RR = 1.25 in 2006 respectively). Social status was also associated with decreased likelihood of remaining single. For instance in 2000, this decreased with increasing status (RR = 0.67, 0.64 and 0.63 for the medium status, rich and richest respectively compared to the poorest). A similar pattern was obtained for 2006, although this was not significant.

### Individual and household characteristics associated with currently married

Among those women who were married compared to others, results indicated that current age, total children born, age at first birth and age at first intercourse were related to marriage. Increase in age was associated with lower probability of marriage in all the years (RR = 0.94, 0.98 and 0.95 for 1992, 2000 and 2006 respectively), while total children born were positively associated with being married. Similarly association was obtained for age at first sex and birth (Table 3).

Significant regional differences for those married were observed in the northeast in 1992 (RR = 1.39, 95% CI = 1.01, 1.95) and in the central region again in 1992 (RR = 0.38, 95% CI: 0.19, 0.75). Having obtained secondary or higher education in 1992 showed a reduced chance of marriage (RR = 0.67, 95% CI: 0.45, 0.99), while results indicated an increased chance of marriage in 2000 (RR = 1.54). Religiosity differences were noted in 2006, with Protestants less likely to be married. Being employed increased the chance of marriage for all the survey years. Similar to the never married, results indicated that social status was likely to reduce the odds of being married. In particular those in the upper social class were less likely to be married, and this was persistent in all survey years (RR = 0.37, 0.15 and 0.16 for the years 1992, 2000 and 2006 respectively). Women who were residing in urban areas compared to being in rural areas had a high probability of being married (RR = 1.59 [95% CI: 1.12, 2.26], 1.72 [95% CI: 1.31, 2.26] and 2.01 [95% CI: 1.57, 2.56] in the year 1992, 2000 and 2006 respectively).

### Individual and household characteristics associated with living together

In the case of women cohabitating, current age significantly reduced the likelihood of cohabitation, whereas total children born, age at first birth, education level and employment status increased the chance of living together. Results indicated that women who attained primary education or secondary and higher education were at increased risk of living together. In fact the risk increased with increased education level. For instance in 1992 the risk increased from 1.45 to 2.44 as one moves from primary to secondary or higher levels of education, and in 2000 the risk changed from 2.19 to 2.68 for the same change in educational level, whereas in 2006 the risk varied between 1.15 and 1.68 for an increased educational level.

## Discussion

There is a clear indication that in any society marriage is dynamic, and Namibia is not an exception (Table 1, Figures 1 to 3). The analysis revealed that marital patterns in a Namibian society is predominantly of never married women, and the rates are increasing, while the proportion of those getting married is falling, nevertheless, these proportions decline as age increases. The proportion of cohabitating couples has remained almost constant, below 20%, for the period 1992 to 2006, but persistently within the same range as those currently married. Comparison with other countries in the region show similarities with South Africa, while for Malawi, Mozambique and Zambia, marriage patterns are dominated by the married group [17], [20].

The inverse relationship of marriage patterns with current age is as expected (Figures 3a–3c, Table 1). As age increases, there is transition from singlehood to marriage or other forms of relationships. Similarly, there is a transition from cohabitation to marriage as age increased. It remains to be explored if such transition differs by birth cohort or marriage cohort. What is clear from Figure 1 is that this transition is much slower in 2006 than earlier years suggesting a period effect. As Steel et al. [12] observed in the British study, patterns of cohabitation into marriage differed by cohort, with the 1950–60 cohort more likely to form marriages than much later cohorts. This was lacking in our study and may be worthwhile to investigate. Their results further indicate that childbearing increased the probability of forming marital union. Since our analysis controlled for number of children ever-born, and the results display a positive association with marriage or cohabitation, we argue that a similar effects as found in the British society are being observed in the Namibian society.

Now much as an increase in age is most likely to lead into marriage, notable, however, is that a plateau of this transition is at 30 to 39 years of age. This differs from other societies in the region, where marriage is entered at a relatively young ager and the plateau occurs much early at 23–27 years. The policy of education for all introduced after independence meant more women became educated, thus delaying marriage [21]–[22]. It is also during this time period that Namibia advocated more about gender equality and women empowerment, encouraging women to exercise their rights including rights to decide when to get married unlike in the past when women were forced into marriage [22]. Evidently delayed transition to marriage has an effect of fertility rate [2]–[3], [12]–[13], nevertheless, the positive relationship with total children born may indicate a catch-up phenomenon within the Namibian society. Be as it may, a total fertility rate of 3.22 shows that late entry into marriage has a big impact.

Our observations further reveal differences in marital status by place of residence, education level and age group. Interesting is a reversal of trends between 2000 and 2006 in all marriage categories. Between 2000 and 2006, there were more singles and fewer married in urban areas, a reverse of what was observed between 1992 and 2000. This is a clear indication that aspects of culture, marriage practices and customs change over time. It seems that this change is rapid in Namibia, especially after gaining stability brought about by attainment of independence in 1990. The effect of urbanization may explain the current trend, but a combined effect of increased educational level could moderate this change in patterns [20], [23]. Historical family studies on in Western European do suggest rapid urbanization, as that experienced in Namibia, does affect marriage patterns. There is a shift in the mean age at marriage, spouse choices change with educated men marrying educated women, and more also other forms of marital status, like cohabitation emerged and singlehood increased [24]. Perhaps, we can argue that the three survey data points: 1992, 2000 and 2006, spanning 14 years is not a long enough series of data to conclude that this is the transition being experienced in Namibia.

The binomial and multinomial analyses indicate that individual and household characteristics contribute significantly towards explaining marriage patterns. The effects of demographic and social differences emerged not only for a single year but were persistent over the years. For example, the effects of total number of children born and age at first intercourse on the singles remained the same, suggesting the non-changing societal attitude towards non-marital child bearing or early sexual interaction being seen as non-traditional behaviour [25]. Different perspectives have merged with regards the timing of first birth. Forms of sexual partnering have been positively related to timing of first birth. Van Roode et al [26] showed that marriage and cohabitation were positively associated with birth timing, a finding which agrees with our results. Similar conclusions were drawn by Steel et al. [11]. However, among singles, early sexual initiation promotes protracted periods of singlehood, and in some cases cause relationship instability at your ages [26].

Education level explains a large proportion of being in singlehood, or marriage or cohabitation. The trend and its effect are similar for the singles and married in that the risk is higher at lower level of education and lower at secondary and higher levels of education. However, the risk of cohabitation is increased with increased education level. Overall, there exists a transformative relationship between education and marital status, with decreasing likelihood of marriage among educated women than the less educated women [27]–[28]. Along with gains in education, womens employment more than doubled over the 10- year period [22]. Employment status, however, has an opposing effect to that of education. Gainful employment solidifies marriage relationships. Being employed increased the chance of being single or marriage or in cohabitation. The effect is similar in all categories in that it generates economic independence, thus a woman can maintain her current status. According to a recent study by Sayer et al. [29], a woman's employment status has no effect on the likelihood that her husband will opt to leave the marriage. An employed woman is more likely to initiate a divorce than a woman who is not employed, only when she reports being highly unsatisfied with the marriage.

With regards regional differences in marriage patterns, our findings suggest that marriage in Namibia is not universal, but display heterogeneity. These results agree with a study done in Mozambique [20], and we attribute such heterogeneous tendencies to variability in ethnic or cultural norms and socio-economic differences. For instance, the central region is more multicultural compounded with the effect of urbanization, while the northeast and northwest are more rural dominated, culture and traditional norms are vital [3]. These disparities may require a multilevel or random effects model that includes regional variables to capture contextual effects. Such models would be an interesting extension to the regression model we fitted here and would be worth exploring.

Religiousness, as an indicator of social control, shows a varying effect among the married and those within cohabitation, and that its effect is changing with time [25]. Of interest is the fact that protestants were less likely to be married that Catholics, as evident in 2006, a fact than has been observed elsewhere, for example in Latin America and USA [30]. The liberal gospel and doctrines as purported by protestants may explain such an association. Vignoli and Ferro [25] argued that Catholic values imposed on Italian society have a positive effect on marriage coherence, compared to north European countries and the USA which have seen rising marital disruption. The effect of religion should be interpreted to have the same effect on marriage patterns as culture has. Beliefs and norms do change over time, and religion has an influence on both of these, which in turn has an impact on marriage practices [17], [20].

In conclusion, our study has demonstrated the fact that demographic and socio-economic characteristics have important and similar effects for all marriage patterns. The role played by these factors is important to inform policy. We are aware that these explanatory factors are limited to explain the complex and dynamic processes that define marriage decisions and practices. However, literature persistently reports on these key factors, and our study has been defined within such general theoretical framework. Moreover, as pointed out by Vignoli and Ferro [25], some of these variables may raise selection bias and endogeneity, and appropriate techniques are required to model the relationship that may exist between our response variable and the explanatory variables. For instance, mixed regression model that incorporates random effects may be appropriate. Random effects may capture some of the unobserved and unmeasured population effects that influence marriage practices. Be as it may, there is an apparent social change in the Namibian society as reflected in the emerging marital patterns.
